# The Impact of Leadership and Employee Satisfaction on the Performance of Vocational College Lecturers in the Digital Era

**DOI:** 10.3389/fpsyg.2022.895346

**Published:** 2022-04-29

**Authors:** H. M. Muhdar, Wahyudin Maguni, Muhtar Muhtar, Bakri Bakri, S. T. Rahma, I. Wayan Ruspendi Junaedi

**Affiliations:** ^1^Institut Agama Islam Negeri (IAIN) Sultan Amai Gorontalo, Gorontalo, Indonesia; ^2^Institut Agama Islam Negeri Kendari, Kendari, Indonesia; ^3^Akuntansi, Universitas Bosowa, Makassar, Indonesia; ^4^Universitas Negeri Gorontalo, Gorontalo, Indonesia; ^5^Universitas Dhayana Pura Bali, Bali, Indonesia

**Keywords:** leadership, employee satisfaction, vocational schools, lecture performance, digital era

## Introduction

Vocational higher education has a strategic function in developing the interests and talents of a nation's generation which, in turn, serves as a benchmark for the future human potential of the country. According to Kadiyono et al. (2020) and Purwanto et al. (2021a,b), management of higher education must be undertaken in a professional, effective, and efficient way in order to respond to the challenges of a competitive era. A leader in this field, therefore, must be someone who is well-qualified, possesses the requisite competence, experience, and has a broad perspective of future organizational development. It is important for an efficient leader to possess the ability to foster a shared spirit among everyone in the organization and build synergies to achieve the vision and mission of the organization. According to Quddus et al. (2020), Sunarsi et al. (2020), and Purwanto et al. (2021a) the level of satisfaction among employees is the key to determining the success of an organization. According to Gelard et al. (2014); Asbari et al. (2021) and Gessler and Ashmawy (2016) employee satisfaction facilitates the management of human resources and fosters enthusiasm, and conversely, if the level of satisfaction is low, it causes problems. According to Hulpia et al. (2011); Suyitno et al. (2014) and Tas (2017) employee satisfaction is a manifestation of a personal commitment that is expressed as a desire to achieve organizational goals, it provides a stimulus to employee performance that is sustainable in the long run. This personal commitment also shapes organizational behavior that boosts the overall performance of the organization. According to Vizano et al. (2020) and Suharto et al. (2022), members who are satisfied with the organization are those that are fully and enthusiastically involved in their work. Another opinion states that employee satisfaction provides a stimulus for employees to be more sympathetic to the organization and inculcates a high sense of loyalty in them, which, in turn, reduces the desire to act unfairly against the organization. It can therefore be concluded that employee satisfaction is very important for every member of the organization.

Similarly, leadership has also become an important factor to consider while addressing several issues relating to the management of employees in an organization, such as a university. There are many instances where leadership succession has become a barrier or an obstacle to the rate of growth in advancing higher education. Leadership here is defined as a process of influencing and motivating a follower or a member in determining organizational goals. According to Kadiyono et al. (2020), and Haudi et al. (2022), leaders always prioritize serving someone or their followers over their leadership status. One of the characteristics of leaders is the ability to develop that potential in others. It was also stated that leaders are people who are willing to serve others by seeking their development and prosperity to fulfill common goals (Indrawan et al., 2020). This study aims to explore how the process of self-satisfaction is established, examine whether a leadership model has been implemented or not, and determine whether it has had an impact on employee performance through employee satisfaction. This research was conducted at a university located in Semarang, Central Java. Researchers used this opportunity to examine all available information and collect data to test whether leadership has a positive and significant impact on the performance of lecturers in higher education; (b) leadership has a positive impact on employee satisfaction among lecturers in universities; (c) employee satisfaction has a positive effect on the performance of lecturers in universities; and (d) employee satisfaction mediates the relationship between leadership and the performance of lecturers in universities. The ideal leadership is one that follows the demands of the Industrial Revolution 4.0, where leaders follow technological developments and have the requisite skills in influencing, encouraging, guiding, directing, and mobilizing those who are responsible for the implementation and development of education and teaching in the digital era (Fahlevi and Alharbi, 2021).

The results of this study showed that employee satisfaction did not mediate the relationship between leadership and lecturer performance. This is because most of the respondents stated that leadership directly influenced the performance of lecturers without employee satisfaction as an intervening variable. Therefore, in this study, leadership played a significant role in influencing the performance of lecturers and, thereby, improved the performance of the organization as a whole. To get a comprehensive view of the results of this study, the following section describes the theoretical framework and the formulation of research hypotheses, research methodology, and research results. Furthermore, an in-depth discussion of the results of the empirical analysis is summarized in the conclusions of the study with suggestions for future research.

## Method

**Figure 1:**
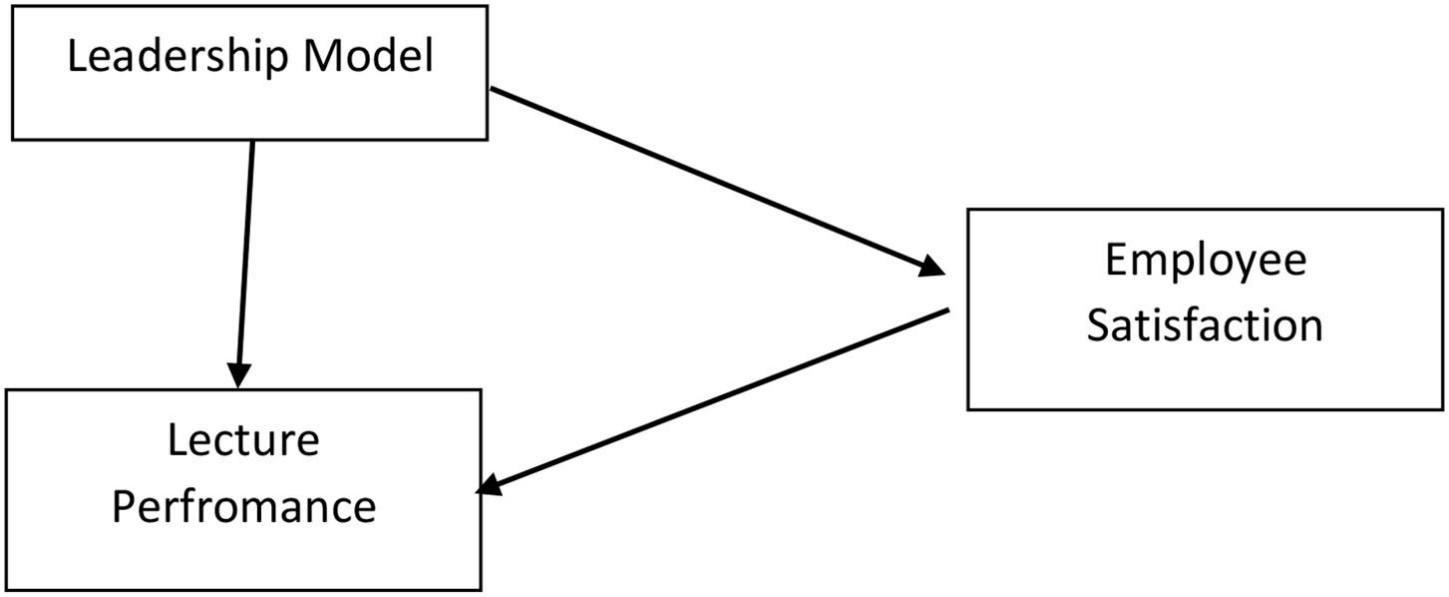
Theoretical framework.

This research is based on a quantitative approach with two independent variables and one dependent variable. The goal is to find the influence of the independent variables, namely leadership (X1) and employee satisfaction (X2), on the dependent variable, lecturer performance (Y). This research was conducted at vocational colleges in Semarang, Central Java. A total of 370 respondents were selected for this study from the scattered population. Simple random sampling was used in this study as it provides equal opportunities to all respondents. Primary data were obtained directly from the respondents through closed online questionnaires, which used a maximum score interval of five and a minimum of one. The research hypotheses that we proposed according Figure 1 in this study are:

H1: Leadership has a positive and significant effect on employee satisfaction of a lecturer in vocational colleges.
H2: Employee satisfaction has a positive and significant effect on the performance of lecturers in vocational colleges.
H3: Leadership has a positive and significant effect on the performance of lecturers in vocational colleges.
H4: Employee satisfaction mediates the relationship between leadership and lecturer performance in vocational colleges.

## Results and Discussion

The validity test used in this study is the Pearson correlation coefficient (also known as Pearson product-moment correlation coefficient). The test results showed that the leadership variable (X1) had a value less than the critical value used, which is 0.05 (α = 5%). This means that all statement relating to the leadership variable is qualified and valid. Employee satisfaction (X2) considered *p* < 0.05 as statistically significant, thereby validating the statement on employee satisfaction. Similarly, the validity test on the lecturer performance variable (Y) was also less than the critical value, which is 0.05 (α = 5%), hence validating the statement on performance. By analyzing the test results of the three variables above, it can be concluded that each statement that represents each research variable is valid. The reliability test results showed that each of the variables studied had a value of more than 0.600, which proves that the instrument analyzed is reliable. Based on these results, the regression equation obtained is as follows:


$$\begin{array}{l} Y=2.405+0.790X1 \end{array}$$

$$\begin{array}{l} Y=-0.601+0.504X1+0.709X2 \end{array}$$

The *t*-test shows that leadership (X1) has a significant effect on employee satisfaction with a positive coefficient and an *R*^2^-value of 0.668, which shows that the ability of the X1 variable in explaining X2 is 66.8%. In the F test, the significance value of *F* is 0.000, which means that leadership and employee satisfaction simultaneously had a significant effect on lecturer performance. This study validates the research by Quddus et al. (2020), Sunarsi et al. (2020), Vizano et al. (2020), Purwanto et al. (2021a,b), and Suharto et al. (2022).

Leadership has a positive and significant effect on employee satisfaction of lecturers at vocational colleges. Employee satisfaction has a positive and significant effect on the performance of these lecturers. Leadership has a positive and significant effect on the performance of the lecturers at vocational colleges. And employee satisfaction bridges the relationship between the leadership model and the performance of lecturers at vocational colleges. These findings confirm the previous studies conducted by Crawford (2003), Birasnav et al. (2013), Desky et al. (2020), Kadiyono et al. (2020), Purwanto et al. (2021a,b), and Haudi et al. (2022).

Path analysis is done by calculating the indirect effect first. Effect is calculated by multiplying the coefficients of the direct influence path traversed. If the result of multiplying the coefficient value is greater than the coefficient that has a direct relationship, then the actual relationship is indirect and *vice versa*. Accordingly, the test results obtained in this study show that employee satisfaction cannot mediate the influence leadership has on lecturer performance as its highest value is still lesser than the direct effect.

## Conclusion

The results of this study show that the type of leadership has a positive and significant effect on the performance of lecturers of vocational colleges. The leadership model has a positive and significant effect on employee satisfaction in vocational colleges. Employee satisfaction has a positive and significant effect on lecturer performance. It could not be proven that employee satisfaction in vocational colleges is able to mediate the relationship between the type of leadership and the performance of lecturers of vocational colleges.

## Author Contributions

HM conceived of the presented idea. WM developed the theory and performed the computations. MM verified the analytical methods. SR encouraged HM and IJ to investigate the topic and supervised the findings of this work. All authors discussed the results and contributed to the final manuscript.

## Conflict of Interest

The authors declare that the research was conducted in the absence of any commercial or financial relationships that could be construed as a potential conflict of interest.

## Publisher's Note

All claims expressed in this article are solely those of the authors and do not necessarily represent those of their affiliated organizations, or those of the publisher, the editors and the reviewers. Any product that may be evaluated in this article, or claim that may be made by its manufacturer, is not guaranteed or endorsed by the publisher.
